# Modern Sociology

**Published:** 1896-10-24

**Authors:** 


### Modern Sociology.

### GOVERNMENT REGULATION OF THE PEOPLE'S FOOD.

Among the most important, if not the most important, of the questions considered by the Select Committee on Food Products Adulteration was that of the establishment of certain standards of purity to which the various sorts of food sold in this country should be made to conform.

At first sight nothing could seem more reasonable than that when a man asks for coffee he should be supplied with nothing but what comes from the coffee bean, and that when he asks for milk he shall get it as it comes from the cow.

The difficulties, however, in the way of formulating any practicable plan by which this may be effected are very considerable, especially if any attempt be made to embody it in an Act of Parliament, the wording of which cannot be altered without the passing of an amending Act, a thing which is by no means easily effected when large trade interests are in question.

For example, it might have been supposed that the definition of the word "food," as used in the Sale of Food and Drugs Act, 1875, was sufficiently comprehensive. Food is there defined as including "every article used for food or drink by man other than drugs or water," and it was not unnatural to assume that this was intended to include all articles which might enter into the composition of food. But a legal decision has been given which shows that this is not the case. It was held that baking powder composed of 20 per cent. of bicarbonate of soda, 40 per cent. of ground rice, and 40 per cent. of alum, was not an article of food, however inseparable from, and essential to, the foods prepared from it it might be, and thus the sale of it, although the alum was injurious, was held not to come under the Act. It is now proposed to amend the Act so as to remove this anomaly; but that is a matter involving considerable difficulty and delay. This, however, leaves untouched the question of food standards.

The Act of 1875 did not so much aim at ensuring the sale of pure food as at preventing the purchaser being supplied to his prejudice with an article whose composition differs from that of the article he asks for, and thus it happens that, whenever the substance asked for is one which varies in composition, the customer gets but small protection—a protection which is reduced to a minimum in the case of those articles the usual adulteration of which consists of the addition of a substance which already exists in it, such as the addition of water to milk.

It seems clear from the evidence offered to the Committee that the present system, by which the Justices or Court dealing with any case are empowered to send samples to Somerset House for official analysis, does not meet the difficulty, in that, however thorough the analysis and complete the opinion may be in any given case, each case is taken by itself, and no attempt is made to fix a standard which might be applicable all round.

Not only so, but it appears that the public analysts consider themselves prejudiced professionally if a certificate given by them is not confirmed on appeal to the Government Laboratory, and thus a strong temptation is placed before them to make themselves safe by not reporting against an article if there is any probability that it would be passed by the authorities at Somerset House on appeal being made to them. Not only, then, are we without any official standard of purity, but our public analysts are prevented from combining to frame one which should be generally accepted by the fact that the Government Laboratory would refuse to recognise it even if drawn up. At present the relations between the Government Laboratory and the public analysts are eminently unsatisfactory, and we cannot but agree with the Committee in regarding it as "of great importance, in the interests of the public, that the public analysts should, as far as possible, be made acquainted with the methods adopted by the Government Laboratory in the analysis of food, and with the considerations kept in view by them in determining whether an article has been adulterated." That a Parliamentary Committee should go out of its way to make so obvious a recommendation shows, at any rate, what the members thought of the relations existing between two sets of public officers, relations far from creditable to the public service.

Not that we would underrate the difficulty of fixing rigid standards of purity in regard to food. If the standard were set low, so as to include the poorest possible unadulterated specimen, the legal definition of the substance in question would be brought down to that low standard. If, on the other hand, it were set too high, the result would be to exclude from the market a large amount of perfectly wholesome food.

The case of milk gives, perhaps, the best illustration of the difficulty of establishing a standard. In New York milk containing less than 3 per cent. of fat is described as adulterated, and in Paris the standard is 4 per cent. But evidence was received by the Committee to the effect that morning milk contained much less fat than that given in the evening, and one witness stated that out of a herd of forty cows in very few cases did the proportion of fat in the morning milk reach 3 per cent., so that were the very moderate standard of 3 per cent. fixed by law a large number of cows would be producing a product which legally would not be milk! Individual cows also vary immensely in the type of milk they give, the proportion of fat ranging between 10·5 and 1·1 per cent.! Clearly the difficulties are very considerable, and we can but agree with the Committee in their recommendation that rather than attempt to fix such matters by Act of Parliament a permanent Court of Reference should be established — a scientific authority which should be empowered to deal, as they arise, with subjects connected with the prescribing of limits and standards of purity in regard to food.

Among the matters to be referred to such a body may be mentioned such questions as the wholesomeness of ingredients used in the preparation of food, and the legitimacy of the use of colouring agents and preservatives. It is also suggested that the proposed court of reference should be entrusted with the duty of collecting and communicating to public analysts and others information as to new and unrecognised forms of adulteration, and of examining and pronouncing upon new methods of analysis. If such a Court were appointed, and if the definition of the word "food," as used in the Sale of Food and Drugs Act 1875, were amended, so as to include expressly all articles intended to enter into or to be used in the preparation or flavouring of food, we might hope soon to see much improvement in the administration of the Acts dealing with adulteration.

